# Supplementary material for: Impact of cyber-invasive species on a large ecological network
Source: Sci Rep. 2018 Sep 5;8:13245. doi: 10.1038/s41598-018-31423-4 (PMC6125364; doi:10.1038/s41598-018-31423-4)
Supplement: Supplementary file 1 — Supplementary Material [file 41598_2018_31423_MOESM1_ESM.pdf]

Online Supplementary Material to: Impact of cyber-invasive species on a large ecological network.

Anna DOIZY* [1], Edmund BARTER[2], Jane MEMMOTT[3], Karen VARNHAM[4] and Thilo GROSS[5]

2018

Contents

1 Methods for collecting the data from the field and the literature **2**

2 Parametrization of the Flat Holm food web **3**

3 Derivation of the sensitivity and influence of the native species **5**

4 Bird groups: generalist and specialist species **8**

References **10**

* author for correspondence (anna@biond.org)
[1] Université Paris-Sud, 91400 Orsay, France and AgroParisTech, 75005 Paris, France
[2] Queen's Building, University Walk, Bristol, BS8 1TR, UK
[3] Life Sciences Building, Tyndall Avenue, Bristol, BS8 1TQ, UK
[4] Royal Society for the Protection of Birds, The Lodge, Sandy, Beds, SG19 2DL, UK
[5] Merchant Venturers Building, Woodland Road, Bristol, BS8 1UB, UK

1 Methods for collecting the data from the field and the literature

The ecological network on Flat Holm, a 35ha island in the Bristol Channel, UK, was constructed from multiple sources (Tab. 1). A study of flower visitor data in 2008 divided the island into four habitat categories: grassland, scrub, buildings (any area with a man-made substrate) and coastal. One hundred minutes of surveying was carried out each day, the time divided between the four habitat types according to the proportion of island surface they covered. Each habitat type was surveyed by walking through it haphazardly looking for insects on flowers. Any insects found during the survey period were caught, preserved and later identified to species level by taxonomists.

Data on the species which are not pollinators or flowering plant come from the South East Wales Biological Records Centre. Interactions between species listed in the biological records are derived from Cramp [1], Pilorge [2], Harde [3], Snow & Snow [4], Luiselli [5] and Skinner [6] and vertebrate-invertebrate scavenger interactions were inferred from Atkinson (unpublished data). Atkinson defines scavenging species as species or genera collected from meat-baited pitfall traps set in mainland south-west England in summer 2012. Due to proximity, many of the same invertebrate species are found on Flat Holm.

Overall, this network contains 560 species (this is not necessarily an exhaustive list of all species on the island) described by 7 sub-networks: plant-Lepidoptera larvae, plant-other invertebrate herbivore, invertebrate-reptile, prey species-bird, plant-flower visitor, plant-seed feeding bird and vertebrate-invertebrate scavenger.

We can divide the links between species in the ecological network into three categories, according to the net gains and losses of each species in the interaction:

1. simple trophic interactions, where one species (the predator) gains and the other (prey species) loses: $+/-$,
2. mutualistic interactions (seed dispersion and pollination) where both species gain: $+/+$,
3. scavenger or parasitic interactions where one species gains (the scavenger or parasite) and the other is, relatively, unaffected (the dead or parasitised species): $+/0$.

**Ecological category**	**No. of links**	**Source**	**Link description**	
Plant/Lepidoptera larvae	643	literature	trophic	$(-/+)$
Plant/Other invertebrate herbivore	9	literature	trophic	$(-/+)$
Invertebrate/Reptile	537	literature	trophic	$(-/+)$
Prey species/Bird	3149	literature	trophic	$(-/+)$
Plant/Flower visitor	375	observation	pollination	$(+/+)$
Plant/Seed-feeding bird	639	literature	seed disperser	$(+/+)$
Vertebrate/Invertebrate scavenger	375	observation	scavenger	$(0\ /+)$

Table 1: Sources of information for the construction of the Flat Holm network

2 Parametrization of the Flat Holm food web

The generalized model is explored by randomly selecting sets of parameters subject to constraints imposed by ecology. The parameters ρ, σ, β, χ, μ depend upon the nature of the feeding relations between the different species, contained in the adjacency matrix. α depends on the inherent characteristics of the species. The remaining parameters ϕ, γ, ψ, λ are chosen from ecologically realistic ranges as contained in Ref. [7] (Tab. 1 in the main paper).

- α_i is the species turnover rate. It is a time-scale parameter which is the same for species belonging in the same taxonomic group. Assuming this parameter is the reciprocal of the life spans of the species, we approximated them as 1 year for the plants (most of them are annual species), 10 years for the birds and reptiles, 0.5 year for the invertebrates and 0.1 year for the fungus.

- ρ_i is the fraction of growth gained by predation. $\rho_i = 1$ for predators and $\rho_i = 0$ for primary producers. Each species gains biomass either by primary production or by predation, but not both.

- σ_i is the fraction of biomass loss resulting from predation. The predation pressure for the top-predators (species without predator) is null, then $\sigma_i = 0$ for them. As we have no more information on the predation pressure for the other species, we assume that $0.5 < \sigma_i < 1$. Values are drawn from a uniform distribution.

- $\beta_{i,j}$ is the relative loss of species j to its predator i among all of j's predators. We assume that the prey j is equally eaten by its predators, so if i eats j then $\beta_{i,j} = 1/$ (number of j's predators) and $\beta_{i,j} = 0$ otherwise.

- $\chi_{i,j}$ is the relative contribution of the prey j to predator i's diet. We assume that the predator i consumes equally all of its prey, so if i eats j then $\chi_{i,j} = 1/$ (number of i's prey) and $\chi_{i,j} = 0$ otherwise.

- μ_i is the elasticity corresponding to the loss of biomass of species i through natural mortality (disease, natural ageing, etc.). In order to stabilize the system, the top-predators have a quadratic mortality rate, i.e. $\mu_i = 2$ which means that there is a quadratic relation between their mortality rate and their biomass X_i. The mortality rate of the other species though increase linearly with their biomass: $\mu_i = 1$.

- ϕ_i is the elasticity corresponding to the gain by primary production. Because of constraints such as space or nutrient availability, the primary production rate is assumed to be sub-linear with respect to the biomass species i. We then chose ϕ_i uniformly between 0 and 1.

- ψ_i is the elasticity corresponding to the gain by predation-ship. Individuals of the same species can interact (either by competition or by mutualism) and this introduces non-linearities in the predation rate. ψ_i is taken uniformly between 0.5 and 1.5; $\psi_i = 1$ would mean that the predation rate is linear with species i abundance, i.e. there is no direct interference between individuals of the same species.

- γ_i is the elasticity corresponding to the gain by predation, but contrary to ψ_i, according to the abundance of i's prey (not i). The availability of prey limits predation, γ_i describes the feeding strategy of species i. As an example, a Holling type II response is close to a linear relation

between the predation rate of i and i's prey abundance ($\gamma_i = 1$). We allow γ_i to be in the range of $[0.5, 1.5]$.

- $\Lambda_{i,j}$ is the elasticity of prey switching. It describes the non-linearity of the contribution of the prey j to the diet of the predator population i. If the predator population does not chose its prey, $\Lambda_{i,j} = 1$. But if the predator adapted a strategy to a specific prey population, $\Lambda_{i,j} = 2$. As we do not know here the strategies of the predator populations, $\Lambda_{i,j} = 1$ or 2 is picked with equal probability (0.5). We note that there is a positive correlation between the proportion of 2s and the stability of the system.

For the full system, we simulated 10^6 Jacobian matrices, 9352 of them lead to a stable steady state, that is $\approx 1\%$. Fig. 1 shows the distribution of the random parameters of the kept simulations. For the simple system with 5 species (described below), 50000 Jacobian matrices were simulated, with $\approx 90\%$ (44833) of them leading to a stable steady state.

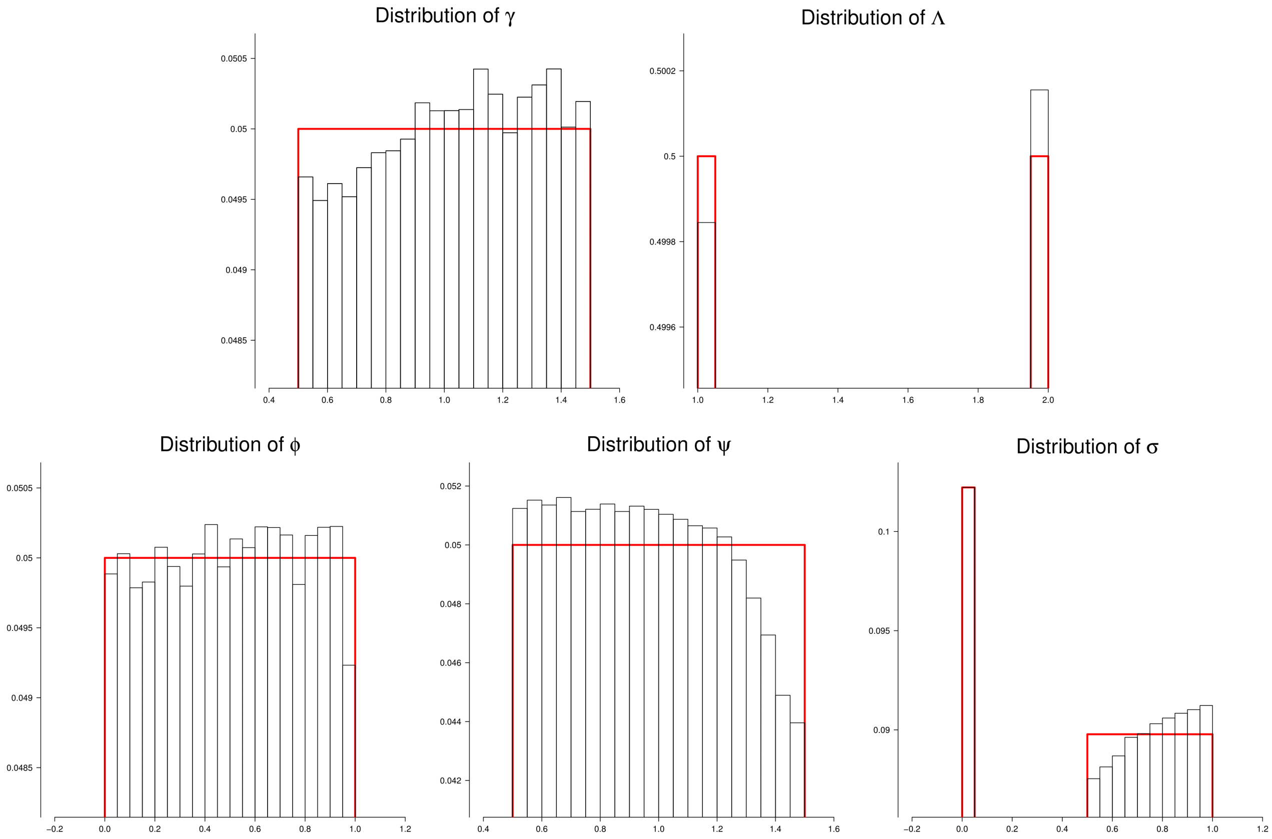

Figure 1: Distribution of the random parameters through the simulations which led to a stable steady state (in black). The red lines are the theoretical distributions of each of the random parameters according to the rules described above. The y-axis is the proportion of kept parameters among the drawn ones.

3 Derivation of the sensitivity and influence of the native species

As a result of the specific structure of the network, different species do not have the same importance within the food web. The Jacobian matrix holds the information relative to each species on how a perturbation of the system at the stable steady state can affect them and how it can propagate trough the network. The strength of the reaction of each species is held by the eigenvalues λ_k. The (right) eigenvectors v_k, that satisfy $\mathbf{J}v_k = \lambda_k v_k$, show the relative amount of each affected species k. Likewise, the left eigenvectors w_k (or the right eigenvectors of $\mathbf{J}^{\mathrm{T}}$), that satisfy $w_k\mathbf{J} = \lambda_k w_k$, show the direction in which the perturbed species k affects the other species through the links of the network. NB: the right and left eigenvalues are the same.

Aufderheide et al. [7] proposed the following definitions for the *sensitivity* and *influence* of species in a food web. The sensitivity Se of a species i represents how it is perturbed by disturbances propagating through the network.

$$\mathrm{Se_i} := \log\left(-\sum_{\mathrm{k}} \frac{|\mathrm{v_k^i}|}{\lambda_{\mathrm{k}}}\right)$$

with v_k the right eigenvector associated to eigenvalue λ_k. The sum is weighted by the invert of the eigenvalues which are relaxation time; so a low λ_k corresponds to a high disturbance in reaction of the perturbation of the kth mode.

The influence Inf of a species i represents how it affects the other species if perturbed itself. It is computed as

$$\mathrm{Inf_i} := \log\left(-\sum_{\mathrm{k}} \frac{|\mathrm{w_k^i}|}{\lambda_{\mathrm{k}}}\right)$$

with w_k the left eigenvector associated to eigenvalue λ_k.

Both sensitivity and influence correspond to intrinsic qualities of the considered populations and their values can be computed directly from the Jacobian matrix. They present a general idea of how a perturbation – e.g. an newly arrived invasive species – can affect the abundances of the native species.

3.1 Confidence regions

Confidence regions are computed to give a guide to distinguish groups of species which are significantly different. The boundary an iso-contour of a Gaussian bi-dimensional distribution and the region contains 95% of all randomly drawn samples. We assume that the data are independent and normally distributed. The origin of the ellipse is placed at the mean of the observations for x and y directions independently. The alignment of the axis of the ellipse are the eigenvectors of the covariance matrix of the data. The lengths of the axis depend on its eigenvalues $a_i^2 = s\lambda_i$, where $2a_i$ is the length of the major-axis ($i = 1$) or minor-axis ($i = 2$), λ_1 and λ_2 represent the sorted eigenvalues of the covariance matrix and the constant of proportionality s is defined as the scale of the ellipse. Using the Chi-square likelihood ($\mathbb{P}(s < 5.991) = 0.95$), the 95% confidence ellipse has the semi-major axis length equal to $\sqrt{5.991\lambda_1}$ and the semi-minor axis length to $\sqrt{5.991\lambda_2}$ (Ref. [8]).

3.2 Results for the full system

We note that:

- The bird-to-bird links are removed to avoid unstable states in the system. Indeed, if the species A eats the species B and B eats A as well, there is a perpetuum mobile and thus energy cannot be produced.

- To achieve stability, no species can have the same prey and predator links, though this is a widely used approximation (niche model [9]). Thus, species with exactly the same prey and predators are assembled in aggregated trophic groups.

The data on the full system can be found on-line [10] in the form of a label adjacency matrix (see definition below).

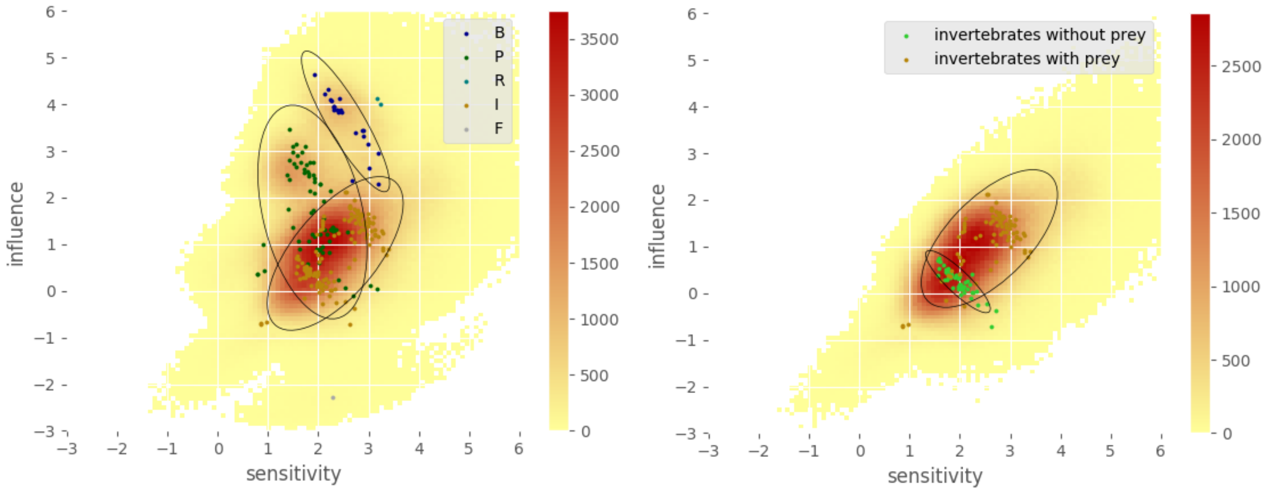

Figure 2: Left: Flat Holm network sensitivity v.s. influence 2D-histogram. Right: Only the invertebrate species. The dots are the sensitivity and influence means for each species on both graphs (**B**irds, **Pl**ants, **R**eptiles, **I**nvertebrates, **F**ungus). The ellipses are the 95% confidence regions for each group of species, *considering only the means of each species.*

Species in the same taxonomic group have similar sensitivity and influence (Fig. 2 Left). The structure of the network, rather than specific values of individual parameters, determines its dynamical behaviour. The influence and sensitivity of the species are not correlated ($R^2 = 0.0196$, $F = 5.518$ on 1 and 225 d.f., $p = 0.02$) and there is a greater variation in influence than sensitivity between species. The birds and reptiles are most influential, with reptiles also showing a relatively high sensitivity to perturbation. Quantities for the plant species show a large variation.

Meanwhile, we can see two different behaviours for the invertebrate group (Fig. 2 Right). A deeper analysis suggests that the invertebrates without prey in the network are amongst the least sensitive and influential ones (figure 2 left). Indeed, the model considers invertebrates without prey as primary producers. As we have only considered simple trophic interactions in the adjacency matrix (and not other interactions such as pollination), some structurally important links are missing from the network.

3.3 Results for the simple system

The species of the Flat Holm ecosystem can be classified into five taxonomic groups: plant (P), invertebrate (I), bird (B), fungus (F) or reptile (R).

The label adjacency matrix $\mathbf{A}$ which gathers the information about the trophic interaction between the species is

$$\begin{array}{c} \\ P \\ I \\ B \\ F \\ R \end{array} \begin{array}{c} \begin{array}{ccccc} P & I & B & F & R \end{array} \\ \begin{pmatrix} 0 & 1 & 1 & 0 & 0 \\ -1 & 0 & 1 & 0 & 1 \\ -1 & -1 & 0 & -1 & -1 \\ 0 & 0 & 1 & 0 & 0 \\ 0 & -1 & 1 & 0 & 0 \end{pmatrix} \end{array}$$

where $A_{i,j} = -1$ if i eats j, $A_{i,j} = 1$ if j eats i and $A_{i,j} = 0$ if there is no interaction between the species i and j. The bird is a top predator, the plant and the fungus are primary producers, the invertebrate feeds on the plant and the reptile feeds on the invertebrate.

Following the rules described in table 1 in the main text and this adjacency matrix, the non-random parameters are

$$\alpha = \begin{array}{c} P \\ I \\ B \\ F \\ R \end{array} \begin{pmatrix} 1 \\ 2 \\ 0.1 \\ 10 \\ 0.1 \end{pmatrix} \qquad \rho = \begin{array}{c} P \\ I \\ B \\ F \\ R \end{array} \begin{pmatrix} 0 \\ 1 \\ 1 \\ 0 \\ 1 \end{pmatrix} \qquad \sigma = \begin{array}{c} P \\ I \\ B \\ F \\ R \end{array} \begin{pmatrix} \sigma_P \\ \sigma_I \\ 0 \\ \sigma_F \\ \sigma_R \end{pmatrix} \qquad \mu = \begin{array}{c} P \\ I \\ B \\ F \\ R \end{array} \begin{pmatrix} 1 \\ 1 \\ 2 \\ 1 \\ 1 \end{pmatrix}$$

$$\beta = \begin{array}{c} \\ P \\ I \\ B \\ F \\ R \end{array} \begin{array}{c} \begin{array}{ccccc} P & I & B & F & R \end{array} \\ \begin{pmatrix} 0 & 0 & 0 & 0 & 0 \\ 1/2 & 0 & 0 & 0 & 0 \\ 1/2 & 1/2 & 0 & 1 & 1 \\ 0 & 0 & 0 & 0 & 0 \\ 0 & 1/2 & 0 & 0 & 0 \end{pmatrix} \end{array} \qquad \chi = \begin{array}{c} \\ P \\ I \\ B \\ F \\ R \end{array} \begin{array}{c} \begin{array}{ccccc} P & I & B & F & R \end{array} \\ \begin{pmatrix} 0 & 0 & 0 & 0 & 0 \\ 1 & 0 & 0 & 0 & 0 \\ 1/4 & 1/4 & 0 & 1/4 & 1/4 \\ 0 & 0 & 0 & 0 & 0 \\ 0 & 1 & 0 & 0 & 0 \end{pmatrix} \end{array}$$

As an example, the plant species has a life-span of one year ($\alpha_P = 1$). It is considered as a primary producer ($\rho_P = 0$). It is eaten as much by the bird as by the invertebrate ($\beta_{\cdot,P} = 1/2$) and its mortality rate is a linear function of its abundance ($\mu_P = 1$). As another example, the life span of the bird is 10 years ($\alpha_B = 1/10$). This species is a predator ($\rho_B = 1$) which eats equally its four prey: the plant, the invertebrate, the fungus and the reptile ($\chi_{B,\cdot} = 1/4$). It is a top-predator ($\sigma_B = 0$), then its mortality rate is a quadratic function of its abundance ($\mu_B = 2$).

We find that the distribution of the influence and sensitivity is multi-modal: the modes correspond to each species of the network (Fig. 3). The multi-modality reflects the structure of the network, whereas the spread of the simulations reflects the variety of systems permitted by the generalized approach.

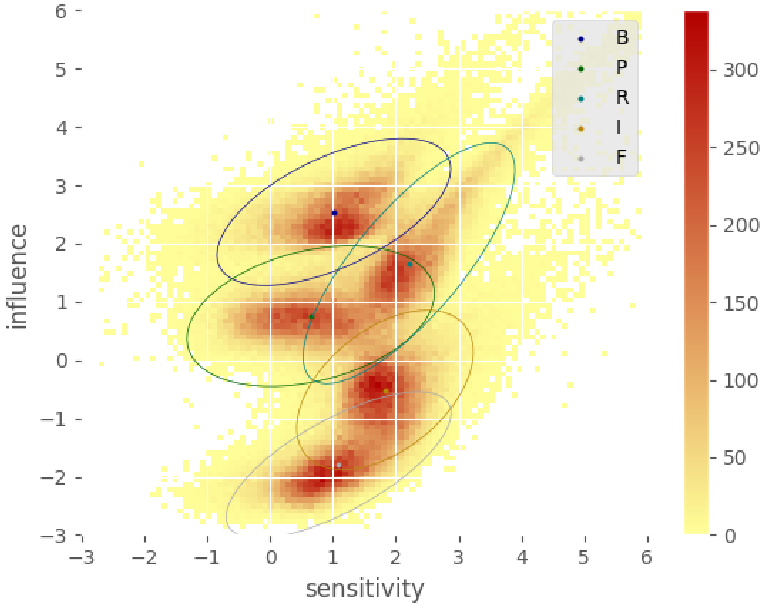

Figure 3: Taxonomic network sensitivity v.s. influence 2D-histogram. The ellipses are the 95% confidence regions for each species, considering *all the simulations*. The dots are the sensitivity and influence means for each species (**B**irds, **P**lants, **R**eptiles, **I**nvertebrates, **F**ungus). The regions are expected to be wider than for the full system, because of the lower variation between the means of the species than between the outcome of the simulations.

4 Bird groups: generalist and specialist species

The birds with the highest sensitivity and lowest influence are positively affected ones by the cyber-herbivore (Fig. 4 Top and Middle). These birds have a lower number of trophic links than the birds with high influence and lower sensitivity (Fig. 5). The cyber-carnivore and cyber-insectivore affect all the birds in similar ways (Fig. 2 in the main paper). In Fig. 4 Bottom, the group of birds which is positively impacted by the cyber-omnivore is not the same one which is positively impacted by the cyber-herbivore. These positively affected birds are those which are not part of the diet of the cyber-omnivore.

The influence of each native species of the Flat Holm food web (apart from the invertebrates) is strongly correlated to its number of trophic links in the Flat Holm network in a non-linear way (Fig. 5). This suggest that generalist species may have larger and more widespread effects on the food webs.

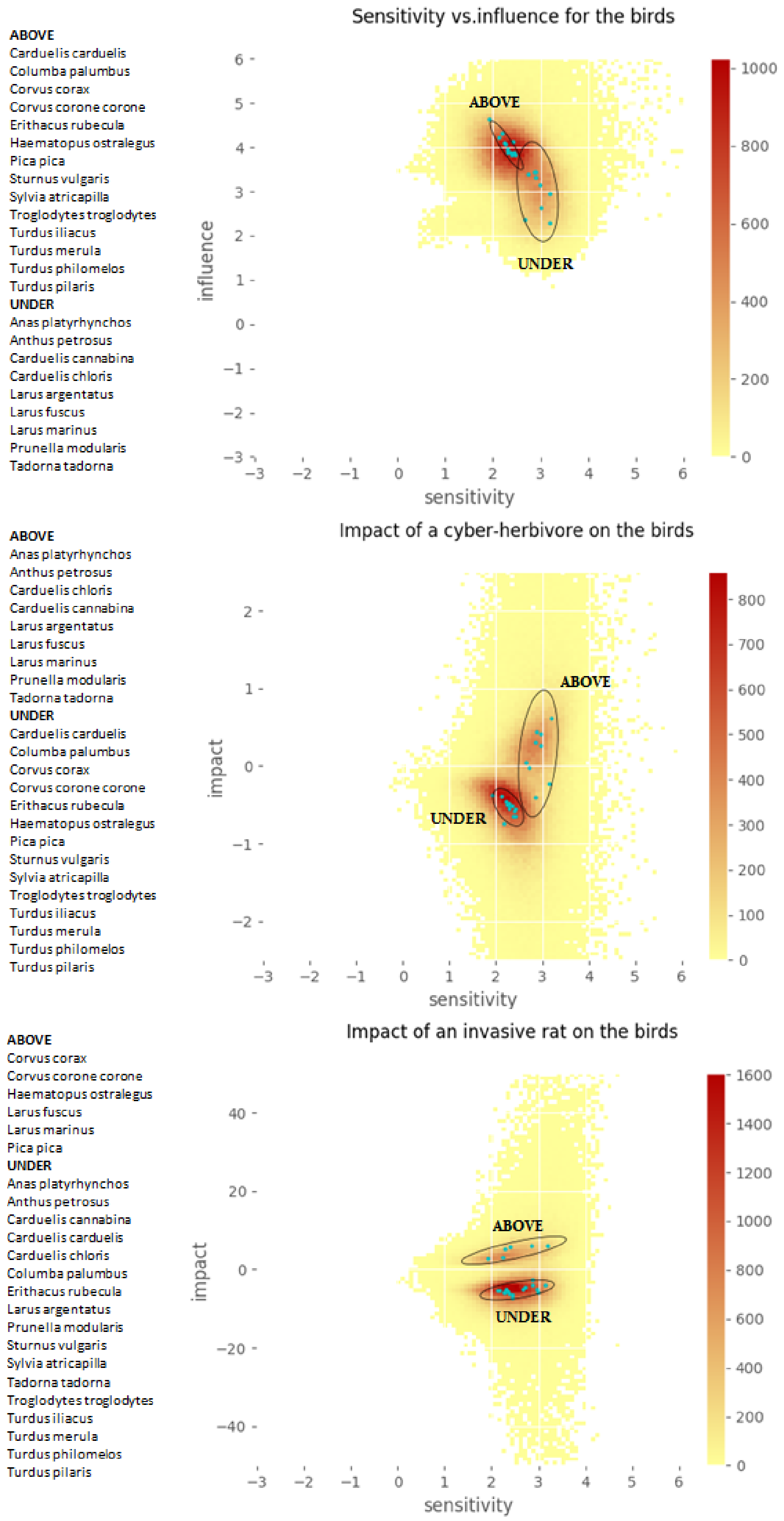

Figure 4: Flat Holm network 2D-histograms for the bird species. The blue dots are the means of the quantities for each species. The confidence regions gather together the species with a similar behaviour. On the left are the details on the species content of each ellipse. Top: Influence v.s. sensitivity. Middle: Impact of the cyber-herbivore v.s. sensitivity. Bottom: Impact of the cyber-omnivore v.s. sensitivity.

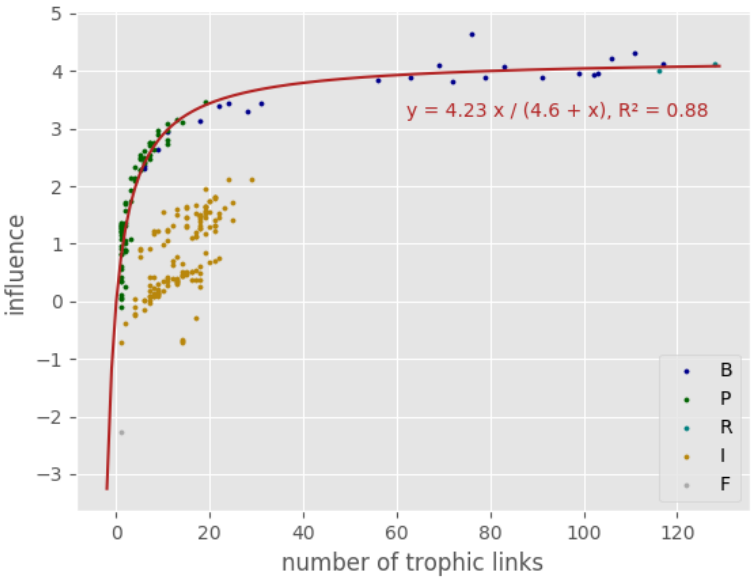

Figure 5: Correlation between the influence of each species (**B**irds, **P**lants, **R**eptiles, **I**nvertebrates, **F**ungus) and their number of trophic links with the rest of the Flat Holm network. The parameters of the non-linear model (in red) are adjusted with a Gauss-Newton algorithm ($p < 10^{-16}$ for both parameters) – the invertebrates are not taken in account in the fit, but are shown on the diagram.

References

1. Cramp, S. *Birds of the western palearctic* Volumes 1-9 (Oxford University Press, UK, 1978-1994).
2. Pilorge, T. Regime alimentaire de Lacerta vivipara et Rana temporaria dans deux populations sympatriques du Puy-de-Dome. *Amphibia-Reptilia* **3,** 27–31 (1982).
3. Harde, K. A Field Guide in Colour to Beetles, Octopus Books, London, 334 pp. (1984).
4. Snow, B. & Snow, D. Birds and berries–A study of ecological interactions. *Poyser, London* (1988).
5. Luiselli, L. The diet of the slow worm, Anguis fragilis Linnaeus, 1758, in the Tarvisio Forest (Carnic Alps, NE Italy). *Herpetozoa* **5,** 91–94 (1992).
6. Skinner, B. *Colour identification guide to moths of the British Isles:(Macrolepidoptera)* (Apollo Books, 2009).
7. Aufderheide, H., Rudolf, L., Gross, T. & Lafferty, K. D. How to predict community responses to perturbations in the face of imperfect knowledge and network complexity. *Proceedings of the Royal Society of London B: Biological Sciences* **280,** 2355 (2013).
8. Johnson, R. A., Wichern, D. W., *et al. Applied multivariate statistical analysis* p. 163 (Prentice-Hall New Jersey, 2014).
9. Williams, R. J. & Martinez, N. D. Simple rules yield complex food webs. *Nature* **404,** 180 (2000).

10. Doizy, A., Barter, E., Memmott, J., Varnham, K. & Gross, T. *Online Supporting Material to: The impact of cyber-invasive species on large, ecologically realistic networks* Content: The label adjacency matrix and perturbation matrix for the full system ; the aggregated trophic groups http://www.biond.org/node/581. 2018.
